# Supplementary material for: Remote transport of high-dimensional orbital angular momentum states and ghost images via spatial-mode-engineered frequency conversion
Source: Nat Commun. 2023 Dec 13;14:8244. doi: 10.1038/s41467-023-43950-4 (PMC10719309; doi:10.1038/s41467-023-43950-4)
Supplement: Supplementary file 1 — Supplementary Information [file 41467_2023_43950_MOESM1_ESM.pdf]

Supplementary information: Remote transport of high-dimensional orbital angular momentum states and ghost images via spatial-mode-engineered frequency conversion

Xiaodong Qiu[1], Haoxu Guo[1], and Lixiang Chen[1,†]

[1]*Department of Physics, Xiamen University, Xiamen 361005, China*

[†]*chenlx@xmu.edu.cn*

Supplementary Note 1. The quality of high-dimensional OAM entanglement

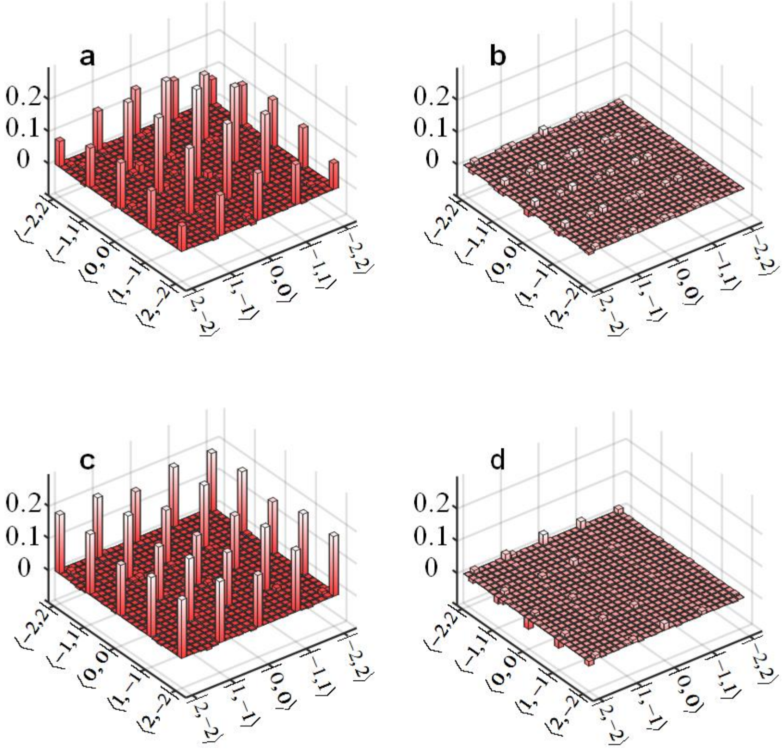

Supplementary Fig. 1. Experimentally reconstructed density matrices for 5-D OAM entangled states. a and **b**: Real and imaginary parts of the density matrix in the LG mode basis, respectively. **c** and **d**: Real and imaginary parts in the perfect vortex basis, respectively.

We employ the generalized Gell-Mann matrix basis[1] to reconstruct the density matrices and use the fidelity[2], $F = \mathrm{Tr}\left(\rho_E \rho_T\right) + \sqrt{1-\mathrm{Tr}\left(\rho_E^2\right)}\sqrt{1-\mathrm{Tr}\left(\rho_T^2\right)}$, to quantify the quality

of entanglement, where ρ_E and ρ_T represent the density matrices of the experimentally reconstructed density matrix and the theoretical ones, respectively. For comparison, we first conduct the quantum tomography of the OAM entanglement in the basis of the standard LG modes, and we present the reconstructed density matrices in Supplementary Fig. 1a and b. We can see that the measured fidelities are only 86.6% and 78.9% for 3-dimensional and 5-dimensional cases, respectively. In contrast, after adopting the perfect vortices as the new OAM basis, we can see from the measured density matrices in Supplementary Fig. 1c and d that the fidelities can be improved to 96.7% and 89.5% for 3-dimensional and 5-dimensional OAM entanglement, significantly surpassing the bounds of 2/3 and 4/5 for 2-dimensional and 4-dimensional entanglement, respectively. Thus we can verify that our generated two-photon states are genuinely entangled in the 3-dimensional and 5-dimensional OAM subspaces, respectively, both of which are fairly suitable for the remote transport of high-dimensional OAM states.

Supplementary Note 2. Error Budget

The main sources of error mainly arise from two-folds: the prepared states (e.g., entangled states and the to-be transferred states) and the imperfect spatio-temporal overlap of coherent beam *a* and photon *b* at BBO2. Regarding the quality of our prepared OAM high-dimensional entangled state, we have measured the density matrices for both the bases of standard LG modes and perfect vortices, respectively, as were shown in Supplementary Fig. 1. Then we have the fidelities of 96.7% and 89.5% for 3-dimensional and 5-dimensional OAM entangled states, which means that the influences of source entanglement on the remote transport of high-dimensional states are roughly ~ 3% (3d) and ~ 10% (5d), respectively. Regarding the quality of the to-be transported states, we prepare several perfect vortices from $\ell = -2$ to $+2$ with the holographic gratings of SLM, and we present in Supplementary Fig. 2 the measured crosstalk matrix. We estimate that the purity of prepared states can reach ~ 97.11%, which means that the effect of the imperfect to-be transported states is ~ 3%.

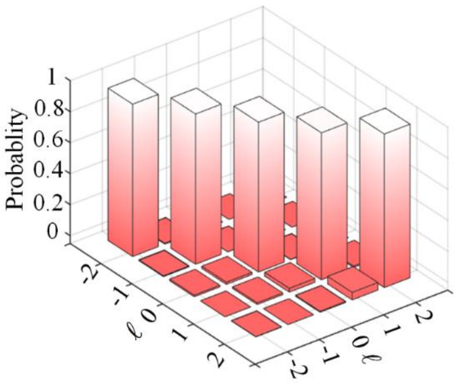

Supplementary Fig. 2. Crosstalk matrix for the prepared perfect vortices from $\ell = -2$ **to** $+2$.

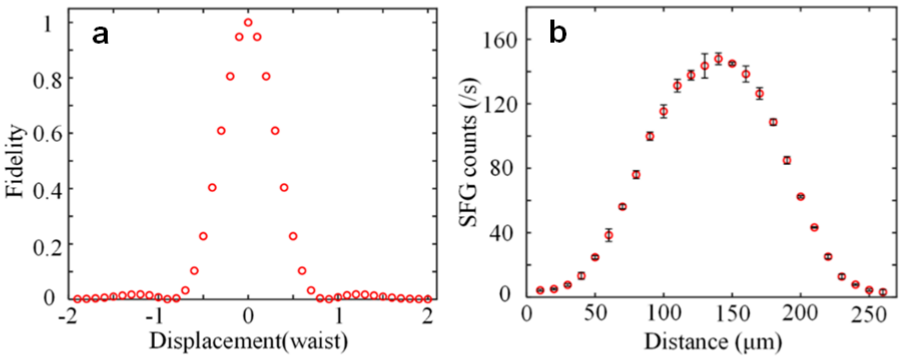

Supplementary Fig. 3. a The fidelity of SFG photons vs. different transverse displacements. **b** The counts of SFG photons vs. different optical path differences.

Regarding the spatio-temporal overlap of coherent beam *a* and photon *b* at BBO2, we first adjust the transverse spatial displacement between coherent beam *a* (e.g., with OAM $\ell = 1$) and photon *b* ($\ell = -1$). Because of OAM conservation, the SFG photon will carry zero OAM $|\ell = 0\rangle$. Then we measure this OAM fidelity of SFG photon, as shown in Supplementary Fig. 3a, which reveals that it is very sensitive to the spatial displacement. In our experiment, we can use the pixel size of SLM ($\sim 12.5\mu m$) to approximate the minimum displacement, and the effect of imperfect spatial overlap is estimated ~ 2%. We then tune the temporal overlap of coherent beam *a* and photon *b* by accurately adjusting the position of right-angle prism (RAP) to introduce different optical path delays. We can see from Supplementary Fig. 3b that the temporal overlap mainly affects the SFG photon counts. While we note that it has no influence on the transfer fidelity.

Supplementary Note 3. Perfect vortices

For making perfect vortices, we need to modulate the standard LG modes to let them bear the same radial intensity profile regardless of carrying different OAM numbers. From the holographic gratings of Supplementary Figs. 4a-4e and the experimental results of Supplementary Figs. 4f-4j, we can see that these modified OAM modes have shared almost the same intensity profile regardless of carrying different OAM numbers, i.e., the perfect vortices.

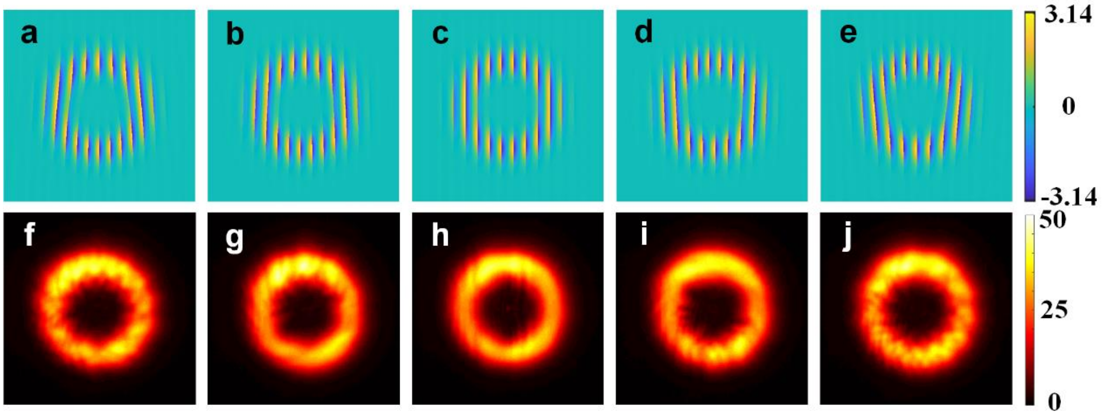

Supplementary Fig. 4. a-e Holographic gratings for preparing perfect vortices with $\ell = -2$ to +2, respectively. **f-j** Experimental observation of doughnut-like intensity patterns for these perfect vortices.

References

1. Bertlmann, R. A. & Krammer, P. Bloch vectors for qudits. *J. Phys. A: Math. Theor.* **41**, 235303 (2008).

2. Mendonça, P., Napolitano, R., Marchiolli, M., Foster, C. & Liang, Y. Alternative fidelity measure between quantum states. *Phys. Rev. A* **78**, 052330 (2008).
